# Development, testing and validation of a targeted NGS-panel for the detection of actionable mutations in lung cancer (NSCLC) using anchored multiplex PCR technology in a multicentric setting

**DOI:** 10.3389/pore.2024.1611590

**Published:** 2024-03-28

**Authors:** Jörg Kumbrink, Melanie-Christin Demes, Jan Jeroch, Andreas Bräuninger, Kristin Hartung, Uwe Gerstenmaier, Ralf Marienfeld, Axel Hillmer, Nadine Bohn, Christina Lehning, Ferdinand Ferch, Peter Wild, Stefan Gattenlöhner, Peter Möller, Frederick Klauschen, Andreas Jung

**Affiliations:** ^1^ Institute of Pathology, Faculty of Medicine, Ludwig Maximilian University of Munich (LMU), Munich, Germany; ^2^ German Cancer Consortium (DKTK), Partner Site Munich, Munich, Germany; ^3^ Dr. Senckenberg Institute of Pathology, University Hospital Frankfurt, Frankfurt, Germany; ^4^ Institute of Pathology, Justus Liebig University Giessen, Giessen, Germany; ^5^ Institute of Pathology, University Ulm, Ulm, Germany; ^6^ Institute of Pathology, University Hospital Cologne, Cologne, Germany; ^7^ Archer, Boulder, CO, United States

**Keywords:** lung cancer, targeted therapy, next-generation sequencing panel, anchored multiplex PCR, actionable mutations

## Abstract

Lung cancer is a paradigm for a genetically driven tumor. A variety of drugs were developed targeting specific biomarkers requiring testing for tumor genetic alterations in relevant biomarkers. Different next-generation sequencing technologies are available for library generation: 1) anchored multiplex-, 2) amplicon based- and 3) hybrid capture-based-PCR. Anchored multiplex PCR-based sequencing was investigated for routine molecular testing within the national Network Genomic Medicine Lung Cancer (nNGM). Four centers applied the anchored multiplex ArcherDX-Variantplex nNGMv2 panel to re-analyze samples pre-tested during routine diagnostics. Data analyses were performed by each center and compiled centrally according to study design. Pre-defined standards were utilized, and panel sensitivity was determined by dilution experiments. nNGMv2 panel sequencing was successful in 98.9% of the samples (*N* = 90). With default filter settings, all but two potential *MET* exon 14 skipping variants were identified at similar allele frequencies. Both *MET* variants were found with an adapted calling filter. Three additional variants (*KEAP1*, *STK11*, *TP53*) were called that were not identified in pre-testing analyses. Only total DNA amount but not a qPCR-based DNA quality score correlated with average coverage. Analysis was successful with a DNA input as low as 6.25 ng. Anchored multiplex PCR-based sequencing (nNGMv2) and a sophisticated user-friendly Archer-Analysis pipeline is a robust and specific technology to detect tumor genetic mutations for precision medicine of lung cancer patients.

## Introduction

Lung cancer, specifically non-small cell lung cancer (NSCLC), covering the large entity of adenocarcinomas of the lung, is a paradigm for precision medicine as there are several tumor drivers known for which targeted drugs have been developed, tested and approved by legal bodies, e.g., EMA (European Medicines Agency), FDA (Food and Drug Administration) and many others [1, 2]. In precision medicine, therapies are patient tailored (personalized medicine) depending on the genetic make-up of the tumor which has to be determined by molecular diagnostics also known as companion diagnostic or theranostic in this context [3–5]. Due to the many known tumor genetic alterations found in lung cancer on the one hand and low amounts of tissue (biopsy, aspirates) on the other hand it is of great advantage to run molecular pathological analyses using multiplexing systems. Thus, NGS (next-generation sequencing) is a good choice and therefore used by many laboratories [6].

In Germany, the national consortium nNGM (national Network Genomic Medicine lung cancer) was established to provide central high quality and continuously advanced diagnostic testing of lung cancer patients from university hospitals, specialized lung clinics and local practices by more than 20 regional university pathology centers. This structure warrants both expertise and up to date molecular pathological diagnostics for the daily care of lung cancer patients [7].

The nNGM follows the strategy to provide a cost-effective analysis of genes/biomarkers that are associated with therapeutic options (EMA approved drugs) or are investigated in German clinical trials which can be reimbursed by German health insurances. However, the reimbursement contract hardly covers the costs for utilization of larger NGS panels. Therefore, a small panel covering mutation hotspots/biomarkers in NSCLC was designed.

The nNGM algorithm includes besides immunostaining of certain markers (e.g., PD-L1) the parallel analyses for single and small multi nucleotide variants on DNA level as well as testing for gene fusions/translocations. Currently, the nNGM guidelines require on the one hand the NGS analysis of pre-defined genomic regions within 26 genes on DNA level. However, the NGS technology of choice is not mandatory. On the other hand, the method of choice for gene fusion/translocation/isoform analyses (*ALK*, *MET* exon 14 skipping, *NTRK1/2/3*, *ROS1*, *RET*) is not stipulated, accepting results from various validated methods including FISH (fluorescence *in situ* hybridization) and NGS (DNA- or RNA-based).

Since different methods, besides NGS, are allowed for gene fusion determination, here we only focus on the cost-effective NGS nNGM DNA panel for the determination of single nucleotide variants, insertions/deletions, duplications and splice site variants. The second version of the nNGM panel (nNGMv2) comprises: *ALK*, *BRAF*, *CTNNB1*, *EGFR*, *ERBB2*, *FGFR1*, *FGFR2*, *FGFR3*, *FGFR4*, *HRAS*, *IDH1*, *IDH2*, *KEAP1*, *KRAS*, *MAP2K1*, *MET*, *NRAS*, *NTRK1*, *NTRK2*, *NTRK3*, *PIK3CA*, *PTEN*, *RET*, *ROS1*, *STK11* and *TP53*.

Different NGS technologies are available for library generation: 1) anchored multiplex- (AMP), 2) amplicon based- (AMPL) and 3) hybrid capture-based (HCP) -PCR [8, 9].

Archer’s VariantPlex technology is based on AMP and has several advantages including low required DNA-amount, high specificity, fast hands-on protocol and easy parallel sample-handling [9]. Moreover, a free sophisticated analysis pipeline is available (Archer Analysis suite, Archer DX). AMP utilizes primer extension and represents an efficient way for specific amplification of DNA (either genomic or cDNA). This technique takes advantage of two different gene specific primers (GSP1, GSP2) per target and allows the amplification of DNA of low quality and or at low amounts [9].

The aim of this multiccentric study was to establish and validate an AMP based cost-efficient NGS panel (ArcherDX VariantPlex nNGMv2), fulfilling the minimum requirements of the nNGM. Therefore, a panel was defined by the nNGM, subsequently designed by ArcherDX and finally tested and validated in a joint effort of four nNGM centers.

## Materials and methods

### Study design and samples

**FIGURE 1:**
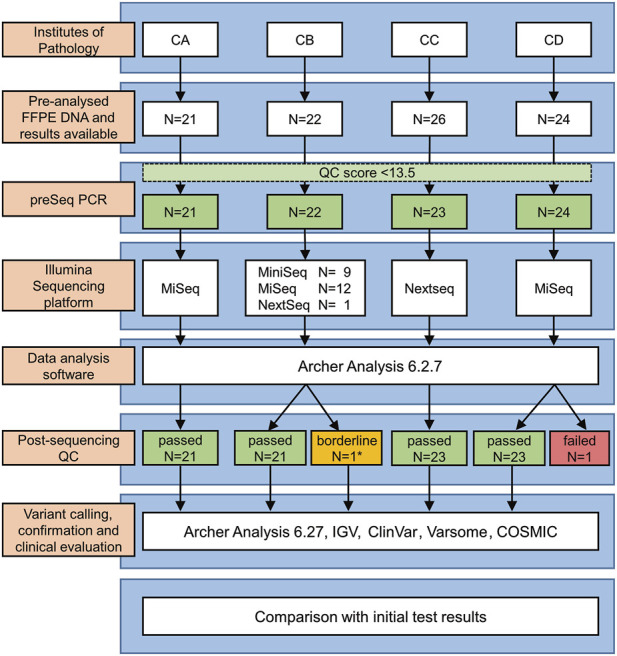
Study design. *Low leftover DNA volume (∼1/3 of the recommended volume).

The study design is depicted in Figure 1. Briefly, the German Institutes of Pathology of the Goethe University (Frankfurt)—center CA, Justus-Liebig University Giessen (Giessen)—center CB, Ludwig-Maximilians-University (Munich)—center CC and University of Ulm (Ulm)—center CD participated in this study. Each center selected 21 to 26 DNA samples (in total 93 samples) from its archives that had been previously analyzed during routine diagnostics by panel sequencing/NGS. DNA had been isolated from formalin fixed paraffin embedded (FFPE) NSCLC tumor samples. The samples were selected to 1) encompass a large repertoire of different tumor genetic variants [SNV (single nucleotide variants); ins (insertions); del (deletions); delins, dup (duplications); splice site variants; rare variants] relevant in NSCLC treatment, 2) comprise a spectrum of low to high DNA concentrations (1–392 ng/μL, median = 23 ng/μL) as well as 3) to cover a broad range of tumor cell contents (2%–90%, median = 50%). The same DNA samples which had been used for the initial routine diagnostics were enrolled in the present validation of our custom ArcherDX VariantPlex nNGMv2 panel (for simplicity mentioned from now on as nNGMv2 panel, ArcherDX, Boulder, CO, United States). In addition, two patient samples (CC-02-28 and CC-03-29) were utilized for serial DNA dilution/reduction experiments for investigating sensitivity and reproducibility. Finally, the commercial Horizon OncoSpan FFPE control (Catalog ID: HD832; Horizon Discovery Ltd., Cambridge, United Kingdom) containing 26 variants verified by NGS and digital droplet PCR of which 13 were covered by our custom panel were analyzed by two centers.

### Histopathological samples and DNA extraction

Leftover DNA isolated from tissue samples of NSCLC patients was used after completion of all diagnostic analyses. Originally, sections from FFPE tissue samples were prepared followed by hematoxylin-eosin (H&E) staining of a representative slide. Areas containing appropriate densities of tumor cells (Supplementary Table S1) were defined and marked by expert pathologists and used as blueprints for transferring marked areas onto unstained serial tissue sections. Marked areas were microdissected under microscopic control and genomic DNA was subsequently isolated using QIAamp DNA Micro Kits (Qiagen, Hilden, Germany; Center CA), Maxwell RSC FFPE Plus DNA Kits (Promega, Madison, WI, United States; Center CB), GeneRead DNA FFPE Kits (Qiagen; Center CC) or Qiagen FFPE AllPrep Kits (Qiagen; Center CD) according to the respective manufacturer’s instructions. DNA concentrations were measured using Qubit3 (Centers CB, CC) or −4 (Centers CA, CD) fluorimeters (Thermo Fisher Scientific, Waltham, MA, United States) (Supplementary Table S1).

### Next-generation-sequencing (NGS) analyses

The routine pre-analyses were performed using Illumina or IonTorrent sequencing instruments together with different panels and analysis pipelines. All analyses used the human reference genome version 19 (hg19) for the alignment and annotation processes. The establishment/validation of the custom nNGMv2 panel analyses were conducted as follows. Briefly, the DNA quality control score (DNA QC Score) of each sample was analyzed using Archer PreSeq DNA QC KITs (ArcherDX) using 5 µL of 1 ng/μL DNA as input. A DNA QC score limit of a maximum of 13.5 was chosen. The recommended DNA input (9.4–359.0 ng; median 102.0 ng) for library preparation based on the QC score was calculated using the PreSeq Calculator.^1^ NGS library preparations were performed according to the Archer VariantPlex Somatic Protocol for Illumina. Library concentration and quality were measured employing Qubit3- (Centers CC, CB) or −4 fluorimeters (Centers CA, CC, CD) (Thermo Fisher Scientific) and 2100 Bioanalyzer (Center CB, CD) or Tapestation instruments (Center CA) (Agilent Technologies, Santa Clara, CA, United States). Subsequently, libraries were sequenced on different Illumina instruments using appropriate flow cells, different library loading concentrations, cluster densities and clusters passing filters. FastQ files were generated from raw sequencing data employing Local Run Manager off-instrument 2.0 (Centers CC, CD; Illumina) and compiled to a single FastQ file per read direction per case by using a self-written python-script (FASTQ Merger v.1.0, CC) or other applications. All further analyses, including quality control and variant calling, were performed applying the cloud-based instance Archer Analysis Unlimited Version 6.2.7. Amongst others, the following sequencing quality metrics were applied: 1) mean target coverage ≥ 100; 2) unique fragment filtered on target percent ≥ 80%; 3) unique fragment total ≥ 150,000; 4) average unique DNA start sites per GSP2 ≥ 50. Analyses that did not pass all four quality limits were evaluated individually by including additional parameters presented in Supplementary Table S2.

### Variant calling, annotation and evaluation

For the initial routine analysis different analysis software and variant calling tools were used. The cut offs for minimum allele frequencies (AF) of previously reported variants were: Center CA 5%, CB 2% (1% at low tumor cell content), CC 3% and CD 5%.

During the validation of the custom nNGMv2 panel the Archer Analysis 6.2.7 default “somatic” filter applying a minimum AF of 2.7% was used. For the detection of *MET* intron variants potentially resulting in *MET* exon 14 skipping a custom filter was used with the following additional settings: Filter Consequence like “intron_variant”; Symbol is *MET*. No filter was used for variants reported in the initial routine analysis with AF below 2.7%.

Variant annotation was performed using the Archer Analysis 6.2.7 as well as the IGV (Integrative Genomics Viewer, Broad Institute, Boston, MA, United States [10]) and the COSMIC (catalogue of somatic mutations in cancer (Sanger-Cancer Center, Cambridge, United Kingdom [11]) database.

Clinical relevance of the identified tumor variants was classified by the standard evaluation algorithm of each center including the ClinVar [12], COSMIC [11] JAX CKB,^2^ OncoKB [13] and Varsome [14] databases/tools. Only likely pathogenic, pathogenic and VUS (variant of unknown significance) variants were reported. Gene variants being a predictive biomarker for EMA or FDA approved targeted therapies were considered “clinically relevant.” Detailed information on identified variants can be found in Supplementary Table S1.

### Statistical analysis

For the correlation of sequencing parameters Spearman’s test was applied using Graphpad Prism 8 (GraphPad Software, Inc., San Diego, CA, United States). Rank correlation values were considered as: very strong relationship: >0.7, strong: 0.40–0.69, moderate: 0.30–0.39, weak: 0.20–0.29. *p-*values lower than 5% (*p* < 0.05, two-sided) were defined as statistically significant.

## Results

### Design and specifications of the custom ArcherDX VariantPlex nNGMv2 panel

The nNGM (national Network Genomic Medicine lung cancer) demands the detection of defined areas including variant hot spots in 26 Genes (*ALK*, *BRAF*, *CTNNB1*, *EGFR*, *ERBB2*, *FGFR1*, *FGFR2*, *FGFR3*, *FGFR4*, *HRAS*, *IDH1*, *IDH2*, *KEAP1*, *KRAS*, *MAP2K1*, *MET*, *NRAS*, *NTRK1*, *NTRK2*, *NTRK3*, *PIK3CA*, *PTEN*, *RET*, *ROS1*, *STK11*, *TP53*). Alterations in these genes are associated with targeted therapies either approved for NSCLC or other solid tumor entities (off-label options) by the EMA, being studied in clinical trials or having other clinical indications (*TP53*). Biomarkers and associated EMA approved drugs or targeted inhibitors are summarized in Table 1.

**TABLE 1:** Covered genes and associated EMA approved drugs or targeted inhibitors.

Gene	Marker	Drugs	Approved/off-label	nNGM trials
*ALK*	Gene fusion/translocation	Crizotinib, Alectinib, Brigatinib, Ceritinib, Lorlatinib	EMA	Yes
*ALK*	Resistance mutation	Brigatinib, Ceritinib, Lorlatinib	EMA	Yes
*BRAF*	V600	Dabrafenib/Trametinib	EMA	
*BRAF*	Activating mutation (non-V600)	RAF/MEK/FAK inhibitors	—	Yes
*CTNNB1*	Activating mutation	—	—	—
*EGFR*	Activating/resistance mutation	Afatinib, Dacomitinib, Erlotinib, Gefitinib, Osimertinib	EMA	Yes
*EGFR*	Exon 20 insertion	Amivantamab	EMA	Yes
*ERBB2*	Activating mutation	Trastuzumab deruxtecan	EMA (CHMP)	Yes
*FGFR1*	Activating mutation	FGFR inhibitors	Off-label EMA	Yes
*FGFR2*	Activating mutation	Pemigatinib, Erdafitinib	Off-label EMA (Pemi.)	Yes
*FGFR2*	Gene fusion	Pemigatinib, Futibatinib, Erdafitinib	Off-label EMA (Pemi./Futi.)	Yes
*FGFR3*	Activating mutation	FGFR inhibitors	Off-label EMA	Yes
*FGFR4*	Activating mutation	FGFR inhibitors	Off-label EMA	Yes
*HRAS*	Activating mutation	MEK/farnesyl transferase inhibitors	—	—
*IDH1*	R132	Ivosidenib	Off-label EMA	—
*IDH2*	Activating mutation	Enasidenib	Off-label FDA	—
*KEAP1*	Inactivating mutation	—	—	—
*KRAS*	G12C	Sotorasib	EMA	Yes
*KRAS*	Activating mutation	MEK/FAK inhibitors	—	Yes
*MAP2K1*	Activating mutation	Cobimetinib, Trametininb	Off-label EMA	—
*MET*	Exon 14 skipping mutation	Capmatinib, Tepotinib	EMA	Yes
*NRAS*	Activating mutation	MEK inhibitors	—	—
*NTRK1/2/3*	Gene fusion/translocation	Entrectinib, Larotrectinib	EMA	Yes
*NTRK1/2/3*	Resistance mutation	Larotrectinib, ALK/RET/ROS1 RTK inhibitors	(Off-label) EMA	Yes
*PIK3CA*	Activating mutation	PI3K/AKT inhibitors	—	—
*PTEN*	Inactivating mutation	—	—	—
*RET*	Gene fusion/translocation	Selpercatinib, Pralsetinib	EMA	Yes
*RET*	Activating/resistance mutation	ALK/RET/ROS1 RTK inhibitors	Off-label EMA	Yes
*ROS1*	Gene fusion/translocation	Crizotinib, Entrectinib	EMA	—
*ROS1*	Resistance mutation	ALK/RET/ROS1 RTK inhibitors	Off-label EMA	—
*STK11*	Inactivating mutation	—	—	Yes (ICI)
*TP53*	Gain/loss of funtion mutation	—	—	—

CHMP, committee for medicinal products for human use; ICI, immune checkpoint inhibitor; RTK, receptor tyrosine kinase.

Notably, for the detection of variants resulting in exon 14 skipping of the *MET* gene coverage of the whole intron 13 and exon 14 as well as 100 bp of intron 14 of the *MET* gene are required to cover also non canonical splice site variants located more distant from the consensus splice sites. Moreover, the nNGMv2 panel covers 12 additional single nucleotide polymorphisms [SNP, rs17793354, rs987640, rs2269355, rs321198, rs338882, rs3780962, rs6444724, rs6811238, rs9951171, rs233214 (X-Chr), rs4829207 (X-Chr), Yp11.2 (Y-Chr)] for genetic identification and verification of tissue (ID-marking) especially in situations of multiple or resistance testing.

Therefore, a specific genomic content (exons, chromosomal regions) was defined on behalf of the nNGM (AH, Cologne) and subsequently converted into the custom nNGMv2 sequencing panel based on the Archer AMP technology by Archer’s design team. This panel includes unique molecular identifiers (UMIs) and was validated by the molecular pathology laboratories of the four nNGM centers University of Frankfurt (center CA), Giessen (CB), Munich (CC) and Ulm (CD). Detailed information on the covered genes, exons/introns and exact chromosomal areas are presented in Supplementary Table S3.

### Study design and validation approach

The study design is depicted in Figure 1. First, each center assembled a list of NSCLCs which were pre-analyzed during routine diagnostics following nNGM regulations. A subset of cases (*N* = 93) was selected encompassing a large repertoire of tumor genetic variants relevant in NSCLC treatment and the majority of genes present in the nNGMv2 panel. Second, DNA quality was determined by qPCR (Archer PreSeq DNA QC, quality score had to be C_T_ ≤ 13.5) allowing *N* = 90 (96.8%) to pass to the third step, the NGS of the samples using the newly designed nNGMv2 panel. MiniSeq (CB), MiSeq (CA, CB, CD) or NextSeq500/550 (CB, CC) sequencing devices (all Illumina) were used (Table 2). Fourth, for reasons of comparability, all results were analyzed applying the same Archer Analysis 6.2.7 software. *N* = 89 (98.9%) of the tested cases passed the Archer analysis pipeline and study internal quality requirements (Table 3, average values from all samples; Supplementary Table S2, values for each sample). Fifth, the resulting variant calls were annotated and evaluated for clinical relevance using not only Archer Suite’s built-in annotation algorithm, but also IGV, COSMIC, Clinvar, JAX CKB, OnkoKB and Varsome. Sixth, in a final step these results were compared to those of the original analyses (Figure 1). Finally, for the determination of sensitivity and robustness of the nNGMv2-panel 1) serial DNA dilution experiments of two cases (center CC) and 2) the Horizon OncoSpan FFPE standard (centers CB and CC) were included, respectively.

**TABLE 2:** Technical parameters.

Center	Nucleic acid extraction method	Cases (N)	Pre-testing				Archer NSCLC panel testing					
			Instrument used	Panel	Cases (N)	Analysis software	Illumina instrument used	Flow cell	Cases (N)	Loading conc. (pM)	Cluster density (K/mm^2^)	Clusters passing filter (%)
CA	QIAamp® DNA Micro Kit (50) (Qiagen)	21	Ion Torrent GeneStudio S5	Thermo Fisher nNGMv2	21	Ion Reporter 5.18.4.0	MiSeq	V3	7	10	972	93.68
								V3	7	10	918	95.13
								V3	7	8	1,008	93.37
CB	Maxwell RSC FFPE Plus DNA Kit (Promega)	22	Illumina MiniSeq/MiSeq	Qiagen nNGMv2	19	CLC Genomics Workbench 20	MiniSeq	Mid Output	5	0.8	232–274	80.77–85.21
			Illumina MiniSeq/MiSeq	Thermo Fisher nNGMv2	3	BaseSpace Variant Interpreter	MiniSeq	High Output	4	0.8	274–309	70.96–84.48
							MiSeq	V2	1	1.7	549	97.81
							MiSeq	V2 Micro	8	1.7	861–1,571	41.40–93.14
							MiSeq	V3	3	1.7	913–1,621	79.81–94.02
							NextSeq	Mid Output	1	1.5	271	71.03
CC	GeneRead DNA FFPE Kit (Qiagen)	23	Ion Torrent GeneStudio S5 prime	Oncomine Comprehensive Assay v3	17	Ion Reporter 5.10 and in-house pipeline	NextSeq	Mid Output	23	1.6	220	87.62
			Illumina Nextseq 500	AmpliSeq forIllumina Comprehensive Panel v3	6	Local Run Manager OFF-Instrument 2.0 and in-house pipeline						
CD	AllPrep DNA/RNA FFPE Kit (Qiagen)	22	Illumina MiSeq	Qiagen nNGMv1	6	CLC Genomics Workbench 5	MiSeq	V2	8	6.5	823	97.42
	GeneRead DNA FFPE Kit (Qiagen)	2	Illumina MiSeq	Qiagen nNGMv2	18	CLC Genomics Workbench 20.0.4		V3	16	7.5	647	98.79

**TABLE 3:** Summary of quality control parameters of all samples.

Parameter	N	Minimum	Maximum	Confidence interval (95%)	Median	Mean	SD (mean)
DNA concentration (ng/µL)	92	1.17	392	29.49–55.3	23	42.4	61.97
DNA QC Score	92	6.64	13.24	9.762–10.31	10.02	10.04	1.321
recommended amount of DNA (ng)	92	7	332	114.9–146.5	116	130.7	75.76
DNA input quantity (ng)	92	9.4	359	99.37–128.9	102	114.1	70.97
Avg. coverage	92	19.61	6,638	2,171–2,730	2,377	2,450	1,350
Coverage 100x	92	0	100	97.9–98.53	98.67	97.3	11.36
Coverage 10x	92	50.67	100	97.71–99.87	100	98.79	5.221
Uniformity (%)	92	91	100	94.94–99.65	99	98.22	1.518
Molbar total num reads	92	7,125	7,051,206	2,215,000–2,937,000	2,019,687	2,575,975	1,744,923
Unique fragment total	92	6,460	2,833,423	828,199–1,060,000	866,423	944,007	559,209
Raw fragment filtered on target (%)	92	84.65	93.7	90.4–91.11	91.09	90.76	1.711
Unique fragment filtered on target (%)	92	79.67	92.25	86.73–87.98	88	87.36	3.016
On target deduplication ratio	92	1	8.92	2.356–3.065	2.155	2.71	1.712
Unique fragment mean length (bp)	92	80.91	217.4	144.8–155.3	152.4	150	25.46
Average unique DNA start sites per GSP2	92	12.72	909	271.6–312.2	297.9	291.9	98.2

### Testing

All participating nNGM centers applied their own standard workflow during routine pre-testing and nNGMv2 analyses starting from nucleic acids extraction and ending with calling/annotation/evaluation of variants (Supplementary Table S1). Each center contributed more than 20 cases (21–26, average 22.5 per center, total *N* = 93, Figure 1 and Table 2) to this validation study. Subsequently, DNA quality was determined with Archer PreSeq DNA QC qPCR kits and the resulting DNA QC scores were transformed into DNA input as described in Materials and Methods. *N* = 90 (96.8%) DNA passed the test and were thus used as an input for NGS library preparation using nNGMv2 kits, sequencing, data processing and cloud-based Archer Analysis 6.2.7 as mentioned in Materials and Methods.

The Archer Analysis software 6.2.7 is available as an 1) on-premise installable virtual machine (VM, UNIX system) or 2) cloud-based instance (Archer Analysis Unlimited Version 6.2.7). In this study, a study-private instance of the cloud-based v6.2.7 was utilized for the analysis of each case. Resulting variants and annotations were revised according to the HGVS (Human Genome Variation Society [15]) nomenclature using IGV and COSMIC, which was especially necessary for variants affecting more than one base pair [multi-nucleotide variants (MNV), del, delins, dup, ins]. As mentioned above, each center evaluated the clinical/biological relevance based on their default classification system. In addition, variants, complying with HGVS rules, from all centers were submitted together with their reference NM-ID (MANE sequence) to ClinVar retrieving their pathogenic potential based on ClinVar evaluation criteria utilizing a NCBI Application Programming Interface (API)^3^ in an iterative manner applying a self-written python-code (PathInfony v.1.4.4, CC) (Supplementary Table S1).

### Quality control (QC)

**FIGURE 2:**
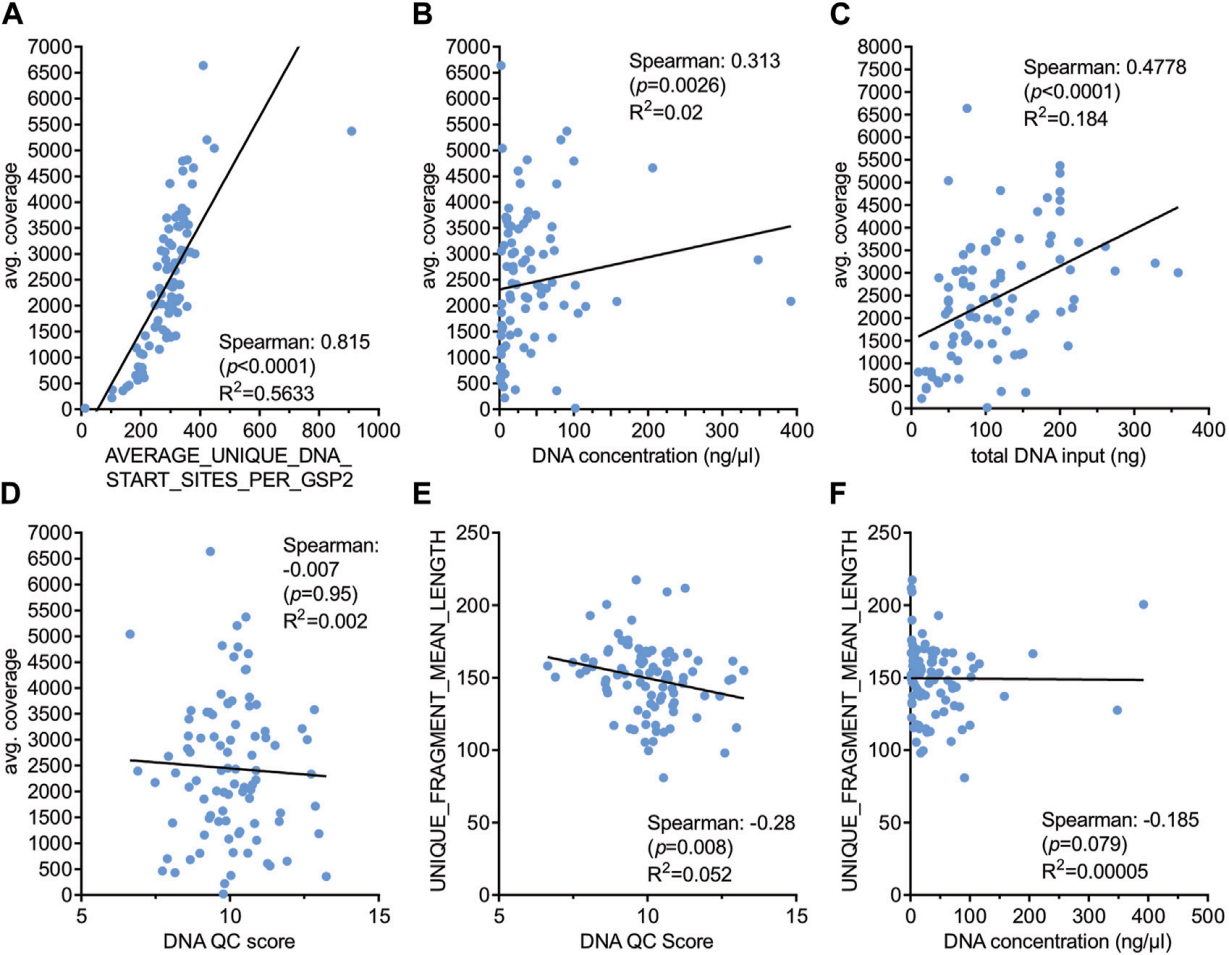
Correlation of various technical parameters with sequencing quality. Spearman correlation was performed with the indicated parameters **(A–F)** and correlation coefficients, *p*-values and *R* ^2^ values are shown. DNA QC score was determined with the Archer PreSeq DNA QC KIT as described in Materials and Methods.

As DNA isolated from FFPE tissue is usually of lower quality compared to fresh tissue it might be important to know the usefulness of individual DNA isolates. Information about the isolated DNA can be obtained from different parameters including DNA concentration measurement and DNA QC scores (Archer PreSeq DNA QC). Parameters indicating the quality of NGS libraries are amongst others *average coverage* and *mean length of unique fragments* of the libraries (Table 3). Expectedly, the *average number of unique start sites per GSP2* (gene specific primer) displayed a very strong correlation with *average coverage* (Spearman coefficient 0.815, *p* < 0.0001, Figure 2A) indicating the usefulness of these parameters given by the Archer analysis pipeline. Neither *DNA concentration* nor *DNA QC score* correlated with *average coverage* (Figures 2B, D) nor *fragment length* (Figures 2E, F). Only *total amounts of DNA* used as input for NGS library preparation showed a strong correlation with *average coverage* (Spearman coefficient 0.48, *p* < 0.0001, Figure 2C).

### Characteristics of the VariantPlex nNGMv2 panel

**FIGURE 3:**
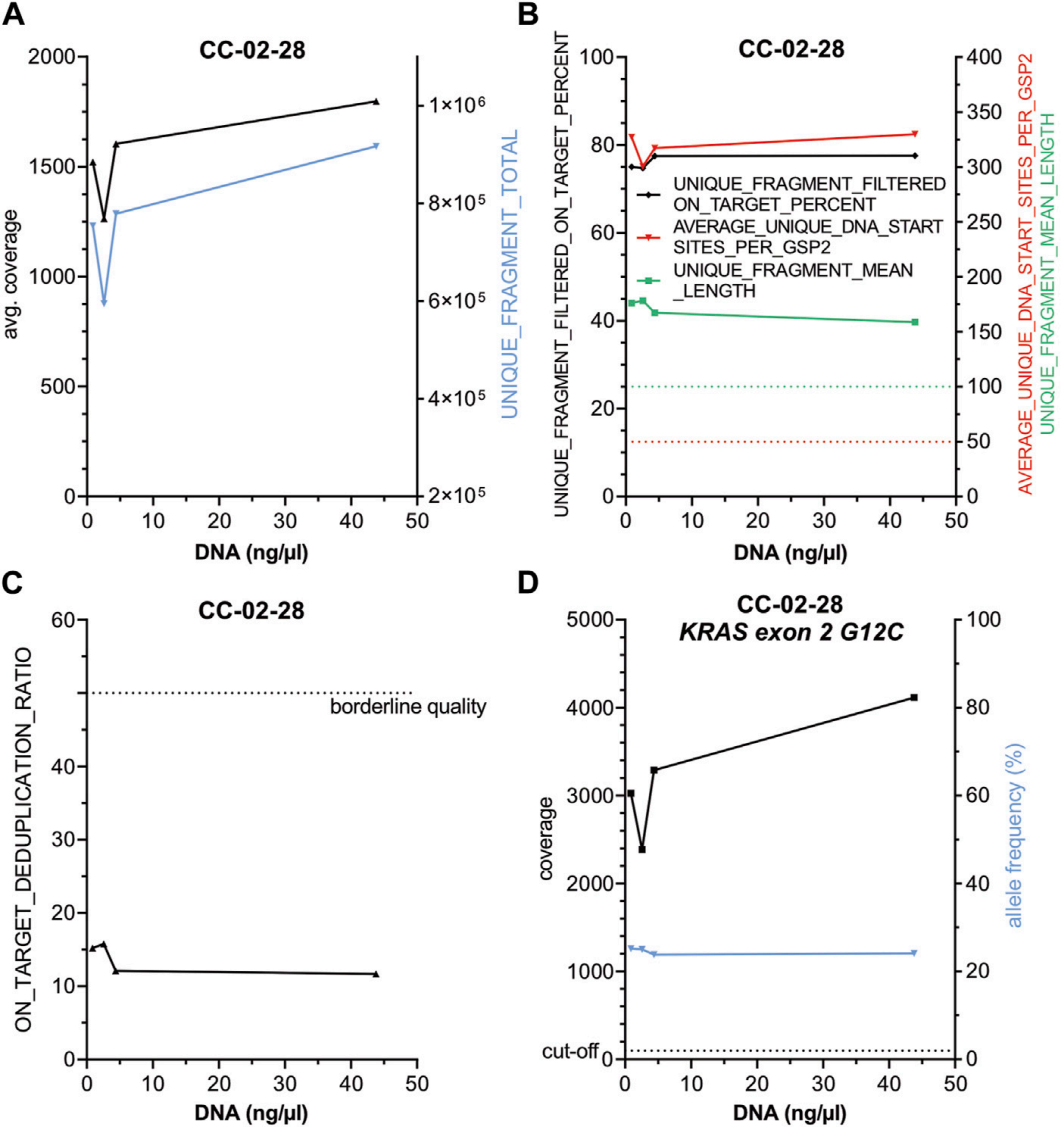
Reduction of DNA concentration does not influence the performance of the nNGMv2 panel. DNA from CC-02-28, harboring a KRAS G12C alteration, was diluted to 44, 4.4, 2.6 and 0.9 ng/μl and similar total amounts of DNA (44–50 ng) were used for library preparation. DNA concentration was plotted against the indicated sequencing quality parameters **(A–D)**. Dashed lines, cut-offs of the shown parameters.

**FIGURE 4:**
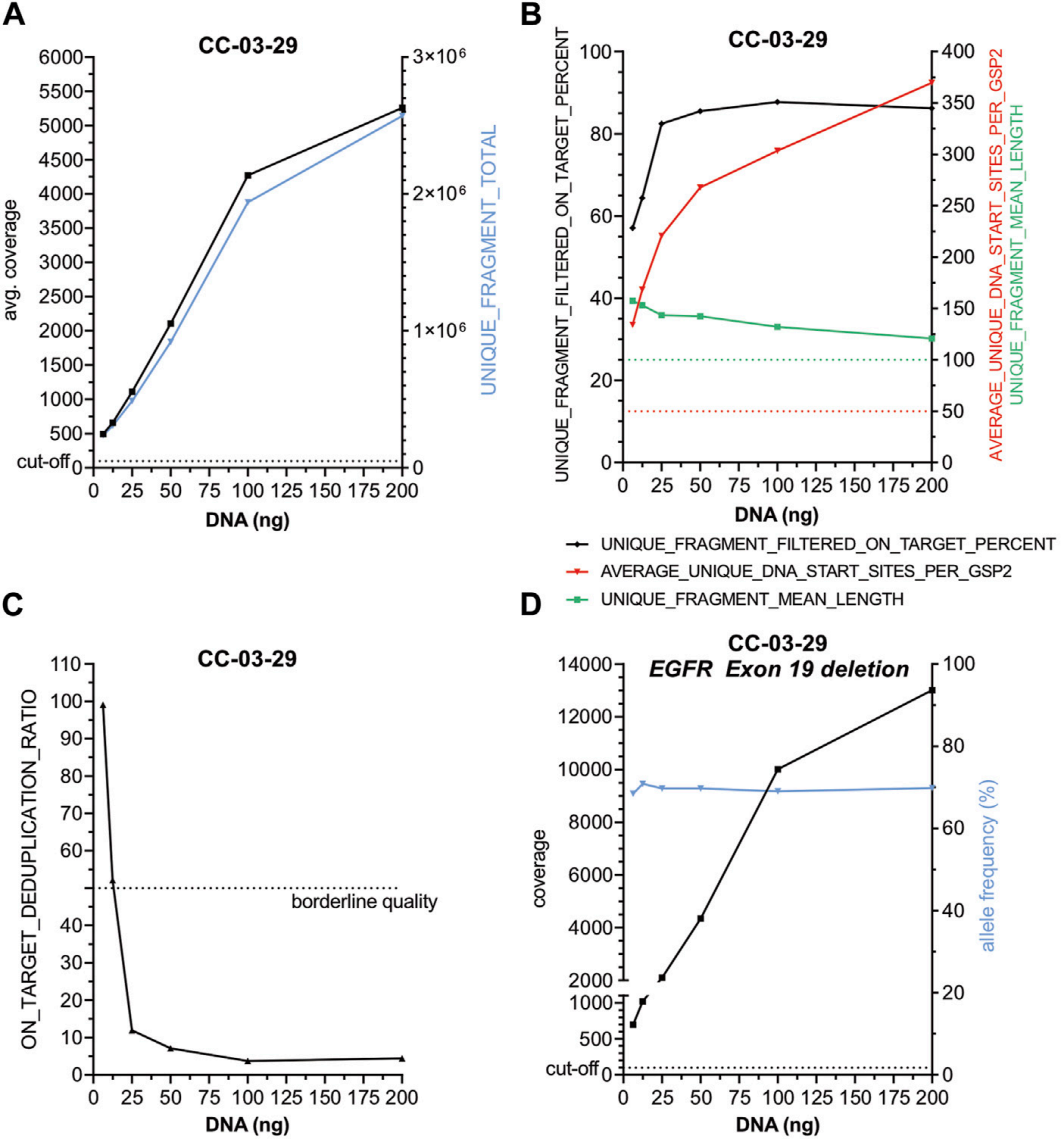
The AMP nNGMv2 panel delivers robust sequencing results with DNA input as low as 6.25 ng. Two hundred, 100, 50, 25, 12.5 and 6.25 ng DNA from case CC-03-29 harboring an EGFR Exon 19 deletion were used for library preparation. DNA amount was plotted against the indicated sequencing quality parameters **(A–D)**. Dashed lines, cut-offs of the shown parameters.

To test whether reduced DNA concentration but similar total DNA input amounts influence the performance of the panel (Figure 3, Supplementary Table S4) a serial dilution of DNA from case CC-02-28, harboring a KRAS G12C alteration, was performed (44.0, 4.4, 2.6 and 0.9 ng/μL) and similar amounts of total DNA from each dilution were used for library preparation. No substantial changes regarding 1) *average coverage* (Figure 3A), 2) on *target deduplication* (Figure 3C) and 3) other parameters (Figure 3B) as well as 4) the KRAS G12C specific coverage and AF (Figure 3D) were observed indicating a high robustness of this panel. Next, a serial reduction of total input DNA (about 200, 100, 50, 25, 12.5 and 6.25 ng) of case CC-03-29 with an *EGFR* Exon 19 deletion was conducted (Figure 4, Supplementary Table S4). As expected, a continuous coverage reduction at lower DNA amounts was observed. However, even at 6.25 ng total input DNA an average UMI coverage of 494 (Figure 4A) and a target specific coverage for the *EGFR* Exon 19 deletion, much higher than the nNGM requirements for UMI panels (∼100), of 697 at similar AF (Figure 4D) were achieved. Expectedly, deduplication rates, an indirect measure for the multiplicity of genomes, increased notably below 25 ng DNA input (Figure 4C). Inversely corresponding, *average unique DNA start sites per GSP2* were greatly reduced below 25 ng DNA to 134 at 6.25 ng but still much higher than the threshold of 50 (Figure 4B).

Taken together, these data indicate that the nNGMv2 panel performance is excellent and stable covering a broad range of DNA input conditions starting with an input as low as an equivalent of 1,000 cells in a reliable manner even in situations where the input material is of lower quality.

### Performance of the nNGMv2 Archer analysis pipeline—testing of a pre-defined FFPE control tissue

In order to evaluate accuracy and specificity of the nNGMv2 panel two centers (CB and CC) subjected the Horizon OncoSpan FFPE control, comprising 13 variants that were cross validated by Horizon with NGS and digital droplet PCR and covered by this panel, to NGS (Supplementary Table S5). All 13 alterations, including three deletions, were identified by both centers and the majority of the variants (80.8%) were detected at AF within the acceptance criteria. Only one variant (PIK3CA E545K) was called by both centers at AF outside the acceptance range (7.00%–10.60%; CB, −3.71%; CC, +18.01%).

### Performance of the nNGMv2 Archer analysis pipeline—comparison with pre-testing results

To investigate the performance, including specificity (variant calling) and precision (AF), of the nNGMv2 panel, the results of the Archer Analysis pipeline were compared with the outcomes of the previous routine diagnostics (*N* = 89; Table 4). QC parameters and variant calling limits as well as the variant evaluation algorithm are described in detail in Materials and Methods. The pre-testing of the cases included different analysis pipelines including IonReporter, Qiagen CLC Workbench and an in-house Illumina pipeline (Tables 2, 4). Sequencing quality of the nNGMv2 panel analyses and of the various utilized Illumina systems are presented in Table 2 per center. The analyzed samples displayed tumor cell contents ranging from 2% to 90% (median = 50%) (Supplementary Table S1). In total, 192 variants were called in the pre-testing analyses in almost all (24—*ALK* and *HRAS* missing) of the 26 genes present in the nNGMv2 panel including 25 deletions (*EGFR*, *KEAP1*, *MET*, *PIK3CA*, *PTEN*, *STK11*, *TP53*), nine deletion insertions (*EGFR*, *FGFR2*, *IDH1*, *KRAS*, *MET*, *TP53*), six duplications (*EGFR*, *KEAP1*, *STK11*, *ERBB2*) and seven splice site variants (*MET*, *TP53*). All alterations but two *MET* intron 13 non-consensus splice variants that might be leading to exon 14 skipping (not confirmed on RNA level) were identified with the Archer Analysis default filter settings. When applying an adapted filter for *MET* intron variants (see Methods) both variants were called. Moreover, three additional variants that were in principle covered by the employed NGS panel (KEAP1 and STK11 truncation variants and a *TP53* point mutation) but not called during pre-testing by the utilized algorithms were identified with the nNGMv2 panel. An excellent precision of the developed panel regarding AF of the variants was also observed. The median AF difference was 0.29% and only 13 variants displayed a difference in AF greater than 10%. In summary of all validation steps, the nNGMv2 panel displayed an excellent specificity, precision as well as robustness. The panel was successfully applied to samples with low cell numbers/DNA content. However, an adapted/second filter was required for the detection of certain more distant non-canonic *MET* exon 14 splice site alterations [16].

**TABLE 4:** Summary: comparison with previous results.

		Previous analyses				Archer analyses (6.2.7)								
Center	Cases (N)	Previous analysis method	Min. % AF^a^	No. of reported variants	Wildtype cases	Successful analyses	Min. % AF^b^	Wildtype cases	No. of reported variants	Discrepant results/variants (%)	Missing clinically relevant variants (targeted therapy)	Missing clinically relevant (targeted therapy) variants with default “somatic” filter	Additional variants	Additional variants clinically relevant (targeted therapy)
**CA**	21	- TF nNGMv2, AmpliSeq 2.0; IonReporter 5.18.4.0	5	36	1	21/21 (100%)	2.7	1	38	2.7	0	0	1 (+1 not covered in previous panel)	0
**CB**	22	- QIAseq nNGMv2; CLC20	2	67	—	22/22 (100%)	2.7	—	68	1.49	0	1**	0 (+1 not covered in previous panel)	0
		- TF nNGMv2 Ampliseq for Illumina; basespace (3 cases)												
**CC**	23	- Oncomine Comprehensive Assay v3; IonReporter 5.10 and in-house pipeline (17 cases)	3	41	2	23/23 (100%)	2.7	2	44	2.5	0	0	1 (+4 not covered in previous panel)	0
		- AmpliSeq forIllumina Comprehensive Panel v3; Local Run Manager OFF- Instrument 2.0.0 and in-house pipeline (6 cases)												
**CD**	24	- Qiagen nNGM V1; CLC5 (6 cases)	5	48	3	23/24 (95.8%)	2.7	2 (+1 wildtype failed)	47	4.26	0	1*	1	0
		- Qiagen nNGM V2; CLC 20.0.4 (18 cases)												
										Sample ID	Gene-ID	HGVS_c	HGVS_p	AF (%)
										*CD-B2-09	MET	c.2942-21_2942-17delinsAGAA	Splicesite	19
										**CB-02-12	MET	c.2942-35_2942-31del	Splicesite	13

AF, allele frequency.

^a^ Reduced at low tumor cell content.

^b^ Default somatic filter.

## Discussion

Here, the AMP-based nNGMv2 panel was established and validated in a multicentric approach for potential diagnostic testing of NSCLC patient samples fulfilling the nNGM analysis requirements [7].

The AMP technique applies sample and molecular (unique molecular identifier) barcode adapter ligation to the DNA followed by usually two subsequent semi-nested PCRs each employing a different gene-specific primer (GSP1, GSP2) in combination with universal primers [9]. AMP offers several advantages compared to AMPL and HCP: 1) amplification is highly specific due to a semi-nested approach with two GSPs reducing off-target amplification, 2) easy multiplexing significantly increasing throughput and cost-effectiveness of NGS library preparation. 3) works with reduced input requirements (both amounts and quality) which is particularly beneficial when working with limited FFPE NSCLC biopsies routinely employed for molecular pathology analyses. 4) high flexibility in target selection making it easy to adapt existing panels, 5) high uniformity of libraries, 6) simple hands-on protocol. Therefore, AMP 7) is easily scalable, 8) has a fast turnaround time as summarized by Zheng et al. [9].

Here, we compare results obtained with the AMP approach (nNGMv2 panel) to results from previous analyses performed during routine testing with AMPL techniques. A direct head-to-head comparison of the techniques was not possible due to lack of sufficient DNA amounts. Nevertheless, both the pre and the AMP nNGMv2 panel testing were performed with the same DNA. Initially, 24–30 samples were selected out of more than 200 recently pre-tested (AMPL) DNAs per center. The samples were selected to encompass various different tumor genetic variants, including complex insertions/deletions and splice site variants, as well as a broad range of DNA concentrations/amounts and tumor cell contents. Moreover, sensitivity (DNA input/cell number) and precision (allele frequency) of the panel were determined by library preparation from serial dilutions of DNA from two cases or a Horizon standard, respectively. We confirmed that the AMP-based nNGMv2 panel in combination with the Archer analysis suite provides specific, reliable and robust results at high uniformity of the sequencing results even with small amounts of input material (DNA input as low as 6.25 ng/∼1,000 cells).

Sequencing was successful in 98.9% of the samples (*N* = 90) even though small biopsies resulting in low DNA concentrations of <5 ng/μL (mean 42.4 ng/μL) in 21% of the samples were included. The sequencing analysis of one sample at a high DNA concentration (102 ng/μL) failed probably due to technical problems. Since all samples were previously tested at least at acceptable quality values and thus preselected, a comparison of both techniques (AMP vs. AMPL) regarding performance cannot be performed. However, after completion of the validation study, Center CC used the AMP-based nNGMv2 panel for routine testing of about 1,000 NSCLC cases until the end of 2022. Initially, extracted DNAs from all FFPE samples were used independent of DNA concentrations for library preparation and subsequent sequencing. Nevertheless, after re-evaluation of the sequencing qualities of the first 100 cases the minimum DNA concentration threshold was set to 2 ng/μL for subsequent cases achieving >96% successful analyses (data not shown). This success rate is high in comparison with the experiences at center CC with various Ion Torrent Oncomine Assays where only 85%–90% of the sequencing analyses were successful at the same DNA concentration cutoff (data not shown). Moreover, it is possible to mix AMP NGS libraries prepared with different techniques, e.g., nNGMv2 or other panels of this type (AMP), Ampliseq for Illumina BRCA- (AMPL) as well as TSO500 HRD-panels (HCP) on a single flow cell in parallel thereby increasing flexibility. Nevertheless, it is recommendable to develop a system specific (Illumina device, flow cell, number of samples) dilution protocol before pooling all libraries as undiluted Archer AMP libraries may lead to irregular clustering depending on the amount of samples.

All but two potential *MET* exon 14 skipping variants were identified at similar allele frequencies with default filter settings in the Archer analysis suite. Both *MET* variants were found with an adapted calling filter showing that, as experienced with analysis pipelines from other sequencing systems, adjustment/refinement of the analysis algorithm is also required to obtain correct/complete results. Three additional variants (*KEAP1*, *STK11*, *TP53*) were called with the nNGMv2 panel that were not identified in the pre-testing analyses. For example, the STK11 truncation variant was not called by the Ion Reporter (5.10) although clearly present in IGV (coverage 1,463, AF 18.9%). Although all three variants do not represent direct targets for approved targeted therapies their detection is important because they might influence the efficacy of immune checkpoint inhibitors in the context of other mutations [17–19]. In this context, our results showed a similar if not even slightly better performance of the Archer analysis suite compared with analysis systems utilized in pre-testing.

It is still an often-discussed question if and how to control quality of input material for generation of NGS-libraries. Several different approaches are available including qPCR measurements of various genes, DNA bio-analyzer profile, DNA ladder amplification and/or DNA quantification [20]. In our study, we demonstrated that using total amounts of DNA as quality determining parameter is superior to a real time PCR based approach (here: Archer PreSeq DNA QC KIT). If this situation is different when applying other qPCR kits or approaches has to be tested experimentally.

Our validation study has clear limitations which are majorly due to the lack of tissue and sufficient DNA amounts as well as the high cost of NGS analyses. 1) For these reasons, a direct head-to-head comparison of the techniques (pre and AMP test) was not feasible, an inter-laboratory exchange (ring trial) was not performed and only limited intra or inter reproducibility tests were included [21]. However, the results obtained with our study design clearly showed the excellent performance of the VariantPlex nNGMv2 panel. Moreover, center CC successfully completed various external ring trials hosted by the Quality in Pathology (QuIP, Berlin, Germany) and the required internal nNGM performance and proficiency tests (including the multigene ring trial) applying the VariantPlex nNGMv2 panel. 2) The VariantPlex system does not cover the detection of gene fusions/translocations and thus is insufficient as a stand-alone biomarker test for NSCLC. Therefore, the nNGM algorithm includes parallel analysis systems for gene fusion detection such as ArcherDx FusionPlex NGS tests or FISH. Recently, ArcherDx expanded the FusionPlex system to perform parallel testing for small nucleotide variants and fusions on RNA level [22]. However, in our hands the coverage of key driver mutations is not optimal, and the corresponding allele frequencies differ significantly from those obtained on DNA level (data not shown). Since the FusionPlex Lung v2 panel is performed in parallel to DNA analysis for detection of relevant gene fusions (ALK, MET, NTRK1/2/2, RET, ROS1), it is rather recommendable for confirmation of variants identified with the VariantPlex analysis.

Taken together, we show that the AMP technology is not only versatile and easy to be used in daily routine diagnostics of NGS-panel based detection of actionable variants in lung cancer but is also a robust technique which can handle DNA of different sources and a broad range of qualities. Thus, AMP represents a suitable workhorse in the daily routine care of molecular pathology.

## Data Availability

The raw data supporting the conclusion of this article will be made available by the authors, without undue reservation.

## Footnotes

1
https://eu.idtdna.com/pages/support/archer-support

2
https://ckb.jax.org/

3
https://www.ncbi.nlm.nih.gov/home/develop/api/
